# Extracellular HMGB1: a therapeutic target in severe pulmonary inflammation including COVID-19?

**DOI:** 10.1186/s10020-020-00172-4

**Published:** 2020-05-07

**Authors:** Ulf Andersson, William Ottestad, Kevin J. Tracey

**Affiliations:** 1 Department of Women’s and Children’s Health, Karolinska Institutet at Karolinska University Hospital, Tomtebodavägen 18A, 171 77 Stockholm, Sweden; 2 Air Ambulance department, Oslo University Hospital, Oslo, Norway; 3 Institute of Clinical Medicine, Faculty of Medicine, University of Oslo, Oslo, Norway; 4 Center for Biomedical Science and Bioelectronic Medicine, Feinstein Institutes for Medical Research, Northwell Health, 350 Community Drive, Manhasset, NY 11030 USA; 5 Donald and Barbara Zucker School of Medicine at Hofstra/Northwell, 500 Hofstra University, Hempstead, New York, 11030 USA; 6 Department of Surgery, North Shore University Hospital, Northwell Health, 300 Community Drive, Manhasset, NY 11030 USA

**Keywords:** COVID-19, SARS-CoV-2, Influenza, Pathogenesis, Pneumonia, ARDS, HMGB1, RAGE, TLR4, Therapy

## Abstract

**Background:**

The 2019 novel coronavirus disease (COVID-19) causes for unresolved reasons acute respiratory distress syndrome in vulnerable individuals. There is a need to identify key pathogenic molecules in COVID-19-associated inflammation attainable to target with existing therapeutic compounds.

The endogenous damage-associated molecular pattern (DAMP) molecule HMGB1 initiates inflammation via two separate pathways. Disulfide-HMGB1 triggers TLR4 receptors generating pro-inflammatory cytokine release. Extracellular HMGB1, released from dying cells or secreted by activated innate immunity cells, forms complexes with extracellular DNA, RNA and other DAMP or pathogen-associated molecular (DAMP) molecules released after lytic cell death.

These complexes are endocytosed via RAGE, constitutively expressed at high levels in the lungs only, and transported to the endolysosomal system, which is disrupted by HMGB1 at high concentrations. Danger molecules thus get access to cytosolic proinflammatory receptors instigating inflammasome activation. It is conceivable that extracellular SARS-CoV-2 RNA may reach the cellular cytosol via HMGB1-assisted transfer combined with lysosome leakage.

Extracellular HMGB1 generally exists in vivo bound to other molecules, including PAMPs and DAMPs*.* It is plausible that these complexes are specifically removed in the lungs revealed by a 40% reduction of HMGB1 plasma levels in arterial versus venous blood. Abundant pulmonary RAGE expression enables endocytosis of danger molecules to be destroyed in the lysosomes at physiological HMGB1 levels, but causing detrimental inflammasome activation at high levels. Stress induces apoptosis in pulmonary endothelial cells from females but necrosis in cells from males.

**Conclusion:**

Based on these observations we propose extracellular HMGB1 to be considered as a therapeutic target for COVID-19.

## Background

Coronaviruses are major pathogens that target the human respiratory system. The recently identified Severe acute respiratory syndrome coronavirus 2 (SARS-CoV-2) may cause pneumonia and primarily infects respiratory epithelial cells utilizing angiotensin-converting enzyme 2 as receptors to gain access to cells (Yan et al. 2020; Lu et al. 2020). Age above 60 years, hypertension, diabetes, and coronary heart disease have been identified as major risk factors for the development of COVID-19, progressing to severe acute respiratory distress syndrome (ARDS) with sometimes lethal outcome (Rothan and Byrareddy 2020; Lake 2020; Huang et al. 2020; Guan et al. 2020). There is experimental evidence that a compromised T lymphocyte-mediated cytotoxic capacity developing during aging mediates the age-dependency of the outcome of the infection. Cytotoxic T cells kill virus-infected cells in a very specific and selective way, causing a silent apoptotic death of the target cell. Void of efficient cytotoxic T cells, innate immunity must try bear the burden of battling the infection. This comes at a high cost. Many more cells will be killed, both virus-infected and bystander cells, and the modes of cell death are highly proinflammatory, involving necrosis, pyroptosis and necroptosis.

There is presently no approved therapies for treating inflammatory mediators in COVID-19 or in other lung injuries. The review is focused on pathogenic mechanisms in lung injury and based on a wide experimental evidence-base. We present possible and highly plausible therapeutic options offered by counteracting selected endogenous proinflammatory mediators. These are mainly released by dying cells and by activated innate immunity cells, both of which we deem central in the pathogenesis of severe pulmonary inflammation. The focus of this review is a possible role of the proinflammatory, endogenous mediator high mobility group box 1 protein (HMGB1) in the pathogenesis of COVID-19.

### Selected endogenous mediators in severe respiratory inflammation

#### High mobility group box 1 protein (HMGB1)

Virally infected or otherwise stressed cells release endogenous damage-associated molecular pattern molecules (DAMPs) to alarm the environment about a loss of intracellular homeostatic balance. HMGB1 is one of the most extensively studied DAMPs and is involved in the pathogenesis of many inflammatory diseases of infectious or sterile origin (Andersson and Tracey 2011; Kang et al. 2014). It is a ubiquitous, evolutionary extremely conserved chromatin-binding protein present in all mammalian nucleated cells and platelets. This 25 kD protein is 99% identical in mammals. The intranuclear functions involve regulation of gene transcription, chromatin repair, and additional tasks. HMGB1 may in addition be passively released extracellularly as a prototypical DAMP from dying cells or actively secreted by stressed or activated cells present in any tissue (Andersson et al. 2018a).

Excessive amounts of extracellular HMGB1 cause release of proinflammatory cytokines including TNF, IL-1 and IL-6 (Andersson et al. 2000). Active HMGB1 release is initiated with a regulated translocation of the nuclear pool of HMGB1 to the cytosol (Bonaldi et al. 2003). Type 1 and type 2 interferons are highly potent endogenous molecules that launch this intracellular relocalization of HMGB1 (Lu et al. 2014; Tanaka et al. 2019). Consequently, administration of interferons as therapeutic antiviral compounds risks to increase extracellular HMGB1 levels, which may promote inflammation rather than mediate beneficial effects. Many chronic inflammatory diseases are characterized by increased circulating HMGB1 levels, possibly of importance for the increased risk of severe outcome in COVID-19 patients with inflammatory comorbidities. Excessive extracellular HMGB1 quantities cause tissue damage and organ dysfunction. In a clinical study, lethality in bacterial pneumonia complicated by ARDS was strongly predicted by initial appropriate antibiotic use and plasma HMGB1 levels (Tseng et al. 2014). Treatment with HMGB1-specific antagonists ameliorates inflammation and improves survival in many preclinical models of acute or chronic inflammatory diseases (Andersson and Tracey 2011; Kang et al. 2014; Andersson et al. 2018b; Yang et al. 2020). However, therapy with HMGB1-specific antagonists has not yet been studied in clinical trials.

HMGB1 receptor usage that generates inflammation is entirely dependent on whether HMGB1 acts on its own or in complex with partner molecules. HMGB1 generally exists in vivo linked to partner molecules. HMGB1 quantification requires that the plasma/serum samples are incubated in acidic buffers prior to antibody-based assessment methods. Steric hindrance precludes antibody detection of HMGB1 epitopes without a proper removal of HMGB1-partner molecules at the initial buffer exposure. HMGB1 has a strong bipolar charge and is prone to complex-bind other proinflammatory molecules including DNA, RNA, histones, nucleosomes, LPS, SDF-1, IL-1α, and IL-1β (Andersson et al. 2018a; Tian et al. 2007; Huang et al. 2016; Deng et al. 2018; Porat et al. 2018). The proinflammatory activities mediated by the HMGB1-partner molecules are amplified in a synergistic manner by HMGB1 (Schiraldi et al. 2012; Wahamaa et al. 2011; Hreggvidsdottir et al. 2009; Sha et al. 2008).

The initial discovery of HMGB1 was based on its ability to bind nuclear DNA (Goodwin et al. 1973). The proinflammatory effects exerted by extracellular HMGB1 were identified at a much later time point (Wang et al. 1999). Experimental studies indicate that HMGB1 plays a critical role in mediating acute lung injury through the recruitment of leukocytes into the lungs (Entezari et al. 2014; Huebener et al. 2015). Furthermore, HMGB1 induced neutrophil dysfunction in experimental sepsis by reducing the NADPH oxidase activity, while treatment with anti-HMGB1 Ab in contrast significantly diminished the sepsis-induced neutrophil dysfunction (Gregoire et al. 2017). Hyperoxia significantly increased the accumulation of HMGB1 in bronchoalveolar lavage fluids prior to the onset of severe inflammatory lung injury (Entezari et al. 2014).

Intratracheal administration of recombinant HMGB1 caused a significant increase in leukocyte infiltration into the lungs compared to animal treated with a control peptide (Entezari et al. 2014). Neutralizing anti-HMGB1 antibodies attenuated pulmonary edema and inflammatory responses, as indicated by decreased total protein content, wet/dry weight ratio, and numbers of leukocytes in the airways (Entezari et al. 2014; Kim et al. 2005). Furthermore, bronchoalveolar lavage fluids from patients receiving long-term mechanical ventilation and ventilator-associated pneumonia have been demonstrated to contain high levels of HMGB1 (van Zoelen et al. 2008). A further understanding of a pathogenic role of local HMGB1 release in pulmonary barotrauma is provided by studies in rabbits exposed to mechanical ventilation with large versus small tidal volumes (Ogawa et al. 2006). Large tidal volumes caused prominent ventilator-induced lung injury, which was associated with 5-fold higher HMGB1 concentrations in bronchoalveolar lavage fluid than those in the low tidal group. Immunostaining of lung tissue showed that alveolar macrophages and infiltrating neutrophils were the major sources of extracellular HMGB1. Interestingly, prophylactic intratracheal installation of anti-HMGB1 antibodies limited microvascular permeability and neutrophil influx into the alveolar lumen and improved oxygenation.

A recent study demonstrated an unexpected gender-specific stress response and HMGB1 release in isolated murine pulmonary endothelial cells (Zemskova et al. 2020). These cells from males were more sensitive than those from females to hypoxia and impaired mitochondrial function to which they responded by necrotic death. Female pulmonary endothelial cells exposed to the same stress preferentially responded by apoptotic cell death. Elevated necrosis was accompanied by significant extracellular HMGB1 release, in contrast to consequences of apoptosis. There is a distinct risk that the increased HMGB1 release in males might contribute to the pro-inflammatory phenotype known to be associated with the male gender.

The number of suggested cognate receptors for extracellular HMGB1 reported in the literature is extensive. However, only two receptor systems, the receptor for advanced glycation end products **(**RAGE) and toll-like receptor 4 (TLR4), are confirmed to act as functional HMGB1 receptors (Rauvala and Rouhiainen 2007; Yang et al. 2010; Yang et al. 2015). Many receptor systems are linked to HMGB1 signaling, however several of them are actually receptors for molecules complex-bound to HMGB1.

#### RAGE

The receptor for advanced glycation endproducts (RAGE) is constitutively highly expressed on alveolar epithelial cells in the lungs, while other tissues show little to no RAGE expression during basic conditions (Brett et al. 1993; Bierhaus et al. 2005; Oczypok et al. 2017). Subsequent studies have localized RAGE expression to the basal membrane of type 1 alveolar epithelial cells, and RAGE has been defined as a specific marker of these cells (Shirasawa et al. 2004; Fehrenbach et al. 1998; Ota et al. 2016). Conflicting results have been reported regarding RAGE expression on type 2 alveolar epithelial cells (Shirasawa et al. 2004; Katsuoka et al. 1997). RAGE will be expressed on vascular endothelial cells and macrophages during inflammatory responses (Brett et al. 1993) (Bierhaus et al. 2005; Oczypok et al. 2017). RAGE was originally identified in diabetes research as a cell surface receptor generating a cascade of intracellular signaling, including nuclear NF-kB translocation and proinflammatory cytokine release (Schmidt et al. 1996). It was later discovered that RAGE is a multiligand receptor and that HMGB1 is one out of many ligands (Rauvala and Rouhiainen 2007). The HMGB1-RAGE axis triggers neutrophil-mediated injury amplification following necrosis (Huebener et al. 2015), something that is of great significance for the pathogenesis of acute lung injury. Interestingly, HMGB1-RAGE interaction does not primarily lead to proinflammatory intracellular signaling. Macrophages expressing RAGE, but engineered to lack TLR4 expression, do not produce proinflammatory cytokines in response to stimulation by HMGB1 of any redox isoform (Yang et al. 2010). That is not the expected result if HMGB1-RAGE interaction mediated cytokine release in a direct mode.

**Fig. 1:**
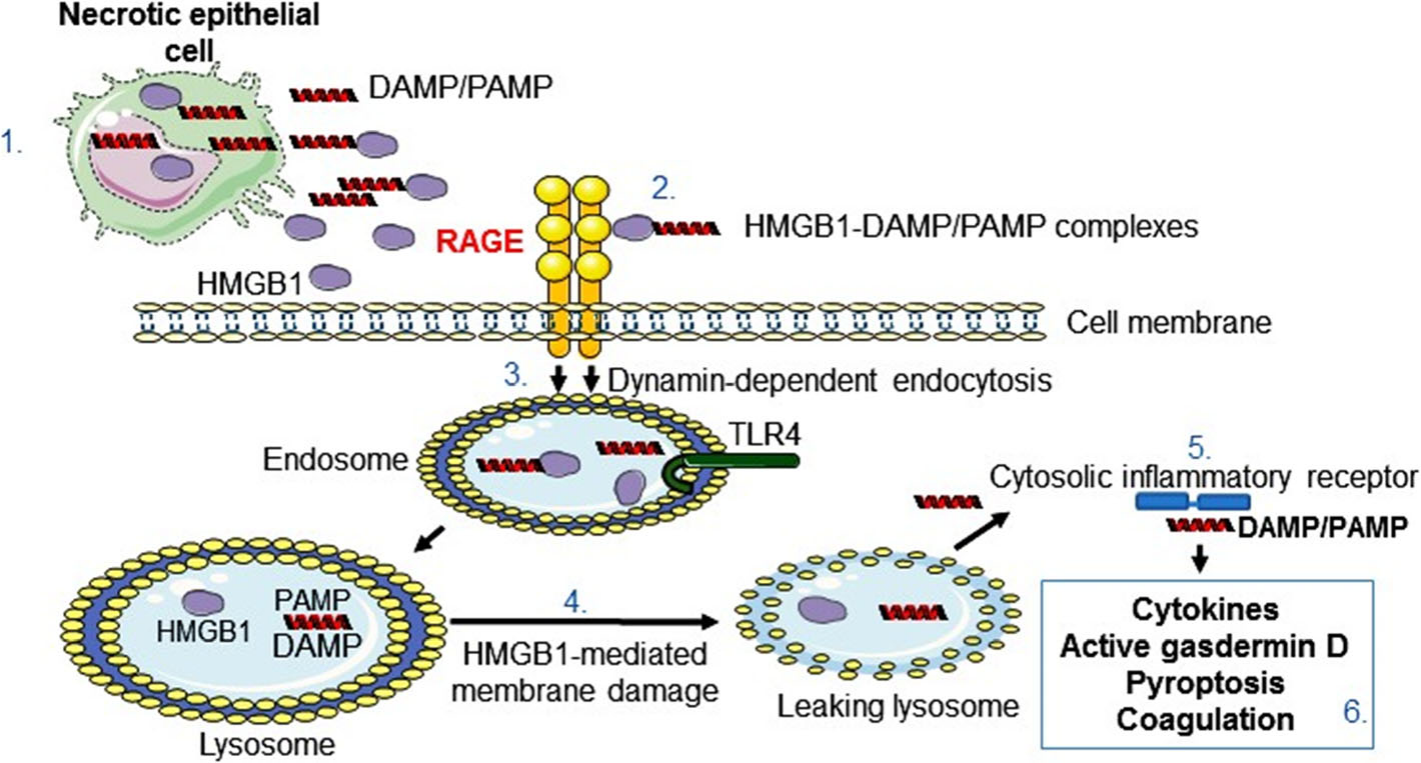
Inflammation induced by HMGB1-partner molecule complexes. Necrotic cells release DAMP and PAMP molecules extracellularly where they form complexes with HMGB1 released from dying or activated cells (1); these complexes bind to RAGE abundantly expressed in the lungs (2); and get endocytosed to endosomes having TLR receptors including TLR4 which may be activated by HMGB1 (3); HMGB1 and partner molecules translocate to lysosomes, where HMGB1 acts like a detergent under the acidic conditions and disrupts the lysosomal membrane enabling HMGB1-partner molecules access to the cytosol (4); the translocated molecules bind and activate reciprocal cytoplasmic receptors generating inflammasome activation and additional proinflammatory events (5); the subsequent outcome production and extracellular release of cytokines via pore formation in the cell surface membrane accomplished by oligomerized gasdermin D. The final outcome is pyroptosis. Active gasdermin D also rotates and translocates phosphatidylserine molecules to the outside of the cell surface membrane and induces tissue factor on endothelial cells. This biology initiates coagulation (6)

Recent observations demonstrate that RAGE provides a transport route for extracellular HMGB1 and HMGB1-partner molecule complexes by endocytosis to the endolysosomal compartment (Deng et al. 2018; Porat et al. 2018; Lin et al. 2019; Yang et al. 2019a; Jia et al. 2019; Xu et al. 2014; Yang et al. 2016a) **(**Fig. 1**)**. The HMGB1/RAGE-assisted cellular import system performs an important task by alerting cells about a dangerous extracellular environment. Most importantly, HMGB1 has a unique ability to act as a detergent in the lysosomal membrane due to the acidic conditions inside the lysosome system (Deng et al. 2018; Xu et al. 2014; Yuan et al. 2020). The HMGB1-transported partner molecules will thus avoid the expected degradation in the lysosomes and instead leak out into the cytosol to reach cognate cytoplasmic receptors, propagating a proinflammatory response. The biological implications of this mechanism may be of tremendous importance for the pathogenesis of severe pulmonary inflammation because the high constitutive cell surface RAGE expression. It has been demonstrated in preclinical and clinical studies that severe respiratory infections including influenza and human respiratory syncytial virus (HRSV) generate substantial extracellular HMGB1 release in pulmonary inflammation and that HMGB1-specific antagonists ameliorate these conditions (Ito et al. 2011; Nosaka et al. 2015; Nosaka et al. 2018; Hatayama et al. 2019; Manti et al. 2018; Rayavara et al. 2018; Rallabhandi et al. 2012; Simpson et al. 2020). Extracellular HMGB1 accumulates locally due to passive release from dying cells and active secretion from innate immunity cells and additional cell types. Furthermore, virus-induced cell death also generates huge quantities of extracellular DNA, RNA, nucleosomes and histones. These molecules are of no major concern as long as they remain extracellularly or get degraded in the lysosomes after cellular import.

Harm may be inflicted if these nuclear danger-molecules will get access to their cognate cytosolic pattern recognition receptors. Excessive amounts of extracellular HMGB1 and abundant cell surface RAGE expression in the tissue enable an intracellular transport of extracellular DNA and RNA, thus providing access to their potent cytosolic cognate receptors cGAS, AIM2,RIG-I and additional nucleic acid receptors with sometimes overwhelming inflammation (Andersson et al. 2018a) (Fig. 1). AIM2 inflammasome activation has been demonstrated to play a key role for influenza-induced lung injury and mortality in preclinical studies (Zhang et al. 2017). AIM2 gene-deficient mice exhibited attenuated lung injury and significantly improved survival with no compromised host antiviral responses.

It should be noted that the biological consequences of HMGB1-RAGE interaction are tissue-specific. While the physiological role of the HMGB1-RAGE pathway in the lungs is elimination of danger molecules carried by HMGB1, the situation is distinctly different in the central nervous system. The original discovery of extracellularly-mediated HMGB1 activity was based on its capacity to mediate neurite outgrowth in the brain via RAGE interaction, without any signs of concomitant inflammation (Merenmies et al. 1991). There are numerous publications on the functional role HMGB1/TLR4-mediated neuroinflammation in brain diseases of both infectious and sterile origin (Ito et al. 2011; Paudel et al. 2018; Nishibori et al. 2019; Frank et al. 2015). COVID-19 also emerges with features of neuroinflammation including fever, loss of small, taste and appetite. We suggest that HMGB1 contributes to these symptoms via TLR4 expression on neurons, microglia cells and astrocytes.

**Fig. 2:**
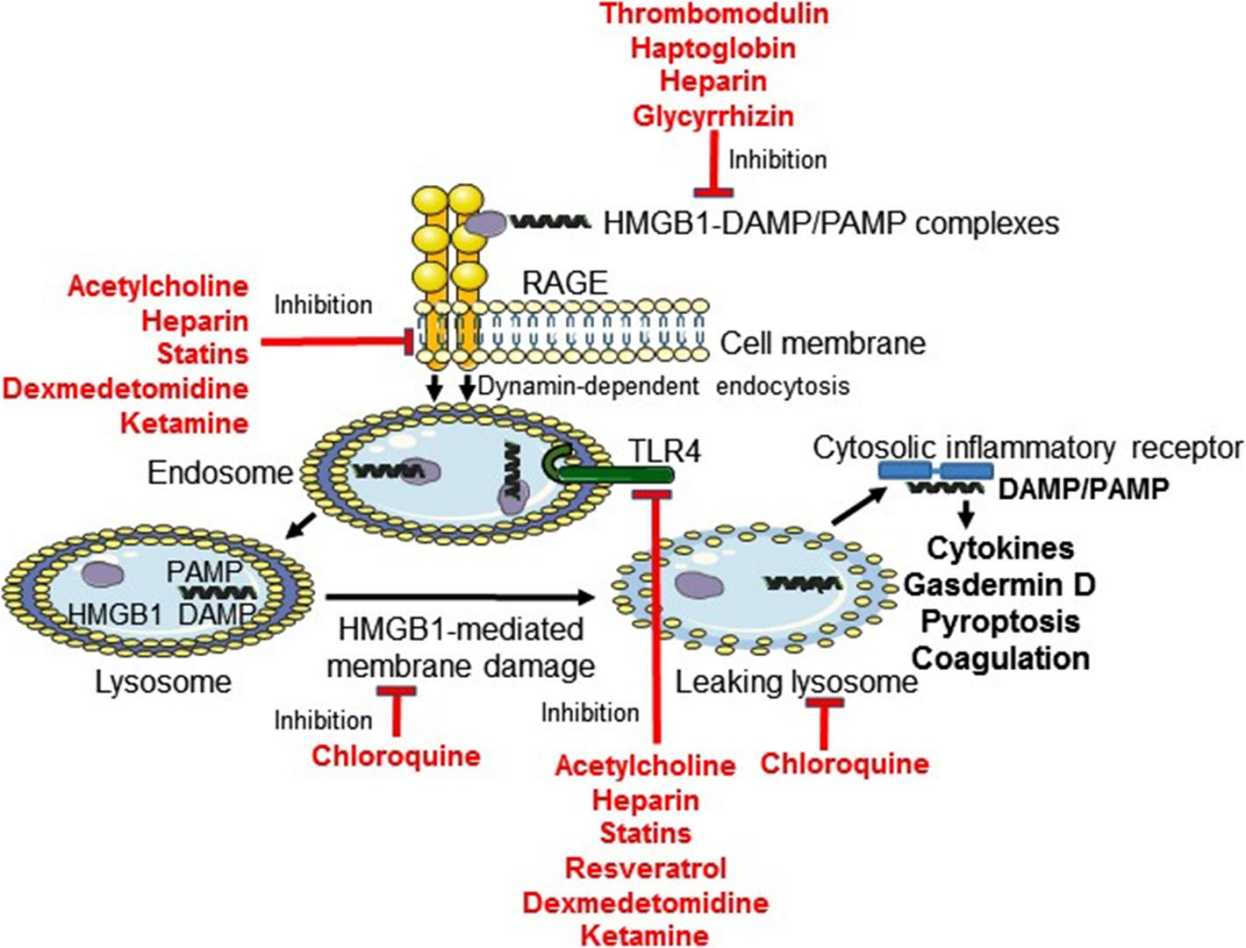
Options to ameliorate HMGB1-mediated inflammation relevant for COVID-19 via pharmaceutical compounds approved for HMGB1-independent indications. The formation of proinflammatory HMGB1-partner molecule complexes is counteracted by thrombomodulin, heparin, haptoglobin, and glycyrrhizin. RAGE-HMGB1-mediated activities are inhibited by acetylcholine, heparin, statins, dexmedetomidine, and ketamine. TLR4-HMGB1 mediated activation is downregulated by acetylcholine, heparin, statins, resveratrol, dexmedetomidine, and ketamine. HMGB1-mediated disruption of lysosomal membrane is constrained by chloroquine phosphate and hydroxychloroquine

Based on the idea that HMGB1 operates as a detergent in the acidic conditions in lysosomes (Deng et al. 2018), it is of distinct interest that a recent clinical study reports that hydroxychloroquine therapy exerts beneficial therapeutic effects in COVID-19 infection (Gao et al. 2020). Chloroquine compounds are known to accumulate in the endolysosomal system and raise the pH, which we hypothesize would counteract HMGB1 from operating as a detergent and thus preclude leakage of DAMP/PAMP molecules to the cytosol (Fig. 2).

A potentially important discrepancy between HMGB1 levels in arterial and venous plasma samples was recently discovered in trauma patients (Ottestad et al. 2019a). Arterial HMGB1 concentrations were consistently lower than venous concentrations in simultaneously obtained samples (arterial = 0.60 x venous; 95% CI 0.30–0.90). Significant amounts of HMGB1 are thus apparently removed in the pulmonary circulation. We doubt that an elimination of HMGB1 itself is the physiological purpose of this function, but rather to degrade PAMP and DAMP molecules carried by HMGB1. The abundant and selective expression of RAGE in the lungs supports extensive endocytosis of HMGB1-partner molecule complexes. These complexes pass the endosomes to accumulate in the lysosomes. We anticipate that physiological amounts of HMGB1 are insufficient to damage the lysosomal membrane allowing the expected degradation of the HMGB1-partner molecule complexes. However pathologically increased intralysosomal HMGB1 levels might cause lysosomal disruption, as already discussed. The leakage sanctions inflammasome activation generating pyroptosis and tissue injury. We suggest that this may be a main mechanism leading to lung injury.

#### TLR4

The redox state of the three cysteines present in HMGB1 is key when HMGB1 acts on its own as a pro-inflammatory DAMP. Gentle oxidation generating a disulfide bond between Cys23 and Cys45 with Cys106 retaining its thiol group forms an HMGB1 redox isoform (disulfide HMGB1) that like lipopolysaccharide (LPS) is a potent functional TLR4 ligand (Kang et al. 2014). Disulfide HMGB1 binds at low nanomolar avidity to the TLR4 co-receptor MD-2, in an analogous way to LPS but at a different position (Yang et al. 2015). Disulfide HMGB1-TLR4 stimulation induces a substantial production of proinflammatory cytokines both in vivo and in vitro (Yang et al. 2010). The clinical outcome of murine influenza infection has been demonstrated to be significantly improved by TLR4-specific antagonists. A small-molecule TLR4-specific antagonist (P5779) that selectively prevents HMGB1-MD-2 interaction, but not LPS from binding to MD-2, protected mice from influenza virus-induced lethality and reduced proinflammatory cytokine gene expression in the lungs (Shirey et al. 2013).

### HMGB1-mediated gene delivery

HMGB1 is a DNA- and RNA-binding protein, a capacity which has been successfully used in cell transfection experiments (Mistry et al. 1997). Plasmid DNA complexed to HMGB1 was demonstrated to be taken up by a variety of mammalian cells in culture. Furthermore, the HMGB1 transfection complexes were not toxic to cultured cells. It is thus conceivable that the RNA of SARS-CoV-2 virus might reach the cellular cytosol through HMGB1-assisted transport, as previously discussed. It is also of interest that a recent clinical pilot study in France indicated that hydroxychloroquine treatment was significantly associated with a reduced SARS-CoV-2 viral load (Gautret et al. 2020). Again, the therapeutic effect might be explained by reduced acidity in the endolysosomal system. The retained viral RNA would then be degraded in the lysosomes.

### HMGB1/RAGE/TLR4 in the pathogenesis of severe pulmonary inflammation

#### Influenza

Influenza viruses, which like SARS-CoV-2 are RNA viruses, cause 3–5 million severe cases and 250.000–500.000 deaths worldwide annually (Paules and Subbarao 2017). These viruses, like the SARS-CoV-2 virus, replicate in respiratory epithelial cells and cause necrotic tissue damage. Influenza-infected patients express elevated circulating HMGB1 concentrations that are associated with the development of severe pneumonia (Ito et al. 2011). Gene-deficient TLR4 as well as gene-deficient RAGE mice are partially protected from influenza-induced lethality (van Zoelen et al. 2009; Nhu et al. 2010). Successful preclinical treatment results using specific HMGB1-, TLR4- or RAGE-antagonists further support that the HMGB1/RAGE/TLR4-axis is central in the pathogenesis of influenza infections. Treatment with anti-HMGB1 mAb provided partial protection against both pneumonia and encephalopathy in murine models of influenza infections despite that the treatments did not affect virus propagation in the lungs (Nosaka et al. 2015; Nosaka et al. 2018; Hatayama et al. 2019). Combined anti-HMGB1 mAb and anti-viral treatment offered almost complete protection against lung injury (Hatayama et al. 2019). Improved survival combined with significantly attenuated histological changes and neutrophil infiltration in the lungs of influenza- inoculated mice have been reported, despite that the treatment was based on xenogenic polyclonal antibodies against HMGB1 (Hou et al. 2014). Therapy with the TLR4-blocking compound eritoran ameliorated murine influenza-induced lung injury by inhibiting the cytokine storm. Additionally, eritoran has been reported to block HMGB1-mediated TLR4-dependent signaling in vitro, and to inhibit extracellular HMGB1 release in vivo by preventing necroptotic cell death in respiratory epithelial cells (Shirey et al. 2013; Shirey et al. 2016).

#### Human respiratory syncytial virus (HRSV)

HRSV is a leading cause of serious lower respiratory tract infection (bronchiolitis and pneumonia) during infancy (Shi et al. 2017), but can also cause severe morbidity and mortality in the elderly and in immunocompromised individuals. HRSV replicates in respiratory epithelial cells and promotes necroptosis and HMGB1 release (Simpson et al. 2020). High HMGB1 levels have been recorded in nasopharyngeal secretion from infected children (Simpson et al. 2020). Experimental RSV infections respond well to certain therapies that exert multiple biological effects, one of which includes HMGB1 antagonism. Two such examples are glycyrrhizin (Manti et al. 2018) and the synthetic TLR4 antagonist eritoran (Rallabhandi et al. 2012).

#### Bacterial pneumonia

Extracellular HMGB1 levels were elevated in all patients with community-acquired pneumonia and higher circulating HMGB1 was associated with disease severity and mortality (Angus et al. 2007; Wang et al. 2017).

Patients with severe pneumonia and ARDS requiring mechanical ventilation experience high rates of ICU mortality. *Pseudomonas aeruginosa* cause neutrophilic lung inflammation in cystic fibrosis patients, who express high HMGB1 levels in bronchoalveolar lavage fluid. Systemic treatment with anti-HMGB1 mAb in a preclinical cystic fibrosis model conferred significant protection against P. aeruginosa-induced neutrophil recruitment, protein leak, and lung injury (Entezari et al. 2012). Treatment with partially desulfated heparin in preclinical models of pneumonia reduced airway HMGB1 levels and neutrophilic lung injury (Griffin et al. 2014; Sharma et al. 2014). Desulfated heparins are derivatives with anti-inflammatory properties but minimal anti-coagulatory effects.

#### Sepsis and coagulopathy

Respiratory failure due to pulmonary hemorrhage is a feared manifestation of disseminated intravascular coagulation occurring in sepsis and other systemic inflammatory disorders. A long-sought-for link between inflammation and excessive activation of the coagulation system has recently been identified in experimental studies of gram-negative bacterial sepsis (Yang et al. 2020; Yang et al. 2019b).

Extracellular LPS derived from the surface of gram-negative bacteria needs to bind to extracellular HMGB1 to be transported via RAGE to the lysosomal system, where HMGB1 enables LPS to escape into the cytosol to bind to its cognate intracellular receptor caspase-11 (the human orthologues are caspase-4 and -5). The binding to caspase-11 causes oligomerization and activation of a complex that converts the pore-forming protein gasdermin D to a cleaved protein promoting the externalization of phosphatidylserine to the outer cell surface via a calcium-dependent phospholipid scramblase (Fig. 1). HMGB1 and LPS also enhance the expression and release of tissue factor that binds phosphatidylserine, which initiates the coagulation cascade. Treatment with anti-HMGB1 mAb in gram-negative sepsis prevented the coagulopathy (Yang et al. 2020). These discoveries have wider implications than gram-negative sepsis biology, since also activated caspase-1, generated by multiple forms of activated inflammasomes, will cleave gasdermin D to a molecule that can activate the coagulation cascade and pyroptosis. The number of DAMPs and PAMPs that activates inflammasomes is considerable.

#### Trauma, shock and reperfusion injury

Experimental work has unambiguously demonstrated a central mechanistic role for HMGB1- mediated injury amplification and pulmonary inflammation in diverse conditions including trauma, shock, and ischemia-reperfusion-injury (Sodhi et al. 2015; Yang et al. 2006; Levy et al. 2007; Shimazaki et al. 2012; Okuma et al. 2012; Kaczorowski et al. 2009). A recent observational study of trauma patients reported that a biphasic release of HMGB1, with a second concentration peak 3–6 h after injury, was a powerful predictor of outcome (Ottestad et al. 2019b). The first peak occurred immediately after the trauma presumably due to a massive necrotic cell death, however not correlated to outcome. The second-wave HMGB1 was a consistent and highly accurate predictor of the duration of the subsequent need for ventilator support, reflecting secondary remote lung injury. Interestingly, second-wave HMGB1 rendered robust predictors like injury severity and physiological derangement (base deficit) insignificant in multivariable prediction models or outcome, possibly acting as a mediator of the combined detrimental effects of anatomical injury and physiologic derangement after trauma. The reasons for the discrepancy between the predictive value of the second versus the initial peak of HMGB1 release are presently not known. We speculate that the two waves of HMGB1 were made up by different HMGB1 redox isoforms as a plausible explanation.

### Options to ameliorate HMGB1-mediated inflammation relevant for COVID- 19 treatment by the use of pharmaceutical compounds approved for HMGB1-independent indications

We find it frustrating that despite two decades of intense exploration of HMGB1 biology, no HMGB1-antagonist has yet reached clinical studies. It is somewhat of a paradox that there are few other conceivable therapeutic target molecules that have been studied in so many successful preclinical inflammatory diseases (reviewed in (Andersson and Tracey 2011; Kang et al. 2014; Andersson et al. 2018b; Nishibori et al. 2019)) and yet it is not presently possible to specifically target HMGB1 in any clinical settings. These preclinical treatment studies have been based on administration of recombinant HMGB1 box A protein for HMGB1 receptor blockade or anti-HMGB1 monoclonal antibodies to inhibit extracellular pro- inflammatory activity via HMGB1 neutralization (Andersson and Tracey 2011; Andersson et al. 2018a; Musumeci et al. 2014). A further clinical development of box A protein has until now been precluded by the lack of in vitro assays evaluating the performance of individual batches. However, this obstacle may now be overcome, since such assays have been accomplished recently (Yang et al. 2019a). Creation of efficient anti-HMGB1 mAbs successfully used in preclinical studies has so far exclusively been contributed by academic research groups. Most of these antibodies are of murine origin, but at least two humanized anti-HMGB1 mAbs have so far been designed (Nishibori et al. 2019; Lundback et al. 2016). A major hurdle for the academia has been a lack of financing enabling clinical studies. The competition around resources to produce and study novel biologics is very challenging.

However, there are already available compounds that have been approved for HMGB1-independent purposes, which might be considered for clinical use to inhibit excessive HMGB1 proinflammatory activities in exaggerated pulmonary inflammation **(**Fig. 2**)**. It should be noted that  these compounds are not HMGB1-specific, but share a capacity to inhibit HMGB1-mediated inflammation in addition to other recognized activities. Whether these other compounds, except from chloroquine and glyccyrhizin, may mediate direct anti-viral effects is currently unproven.

#### Chloroquine

Chloroquine phosphate-based therapy in China and hydroxychloroquine treatment in South Korea have been reported to improve outcome in COVID-19 infections (Gao et al. 2020), but larger controlled studies are still missing. There are several studies indicating that chloroquine compounds exert direct anti-viral activities. Vincent et al. reported that chloroquine prevented the spread of the SARS coronavirus in cell culture by interfering with terminal glycosylation of the cellular receptor, angiotensin-converting enzyme 2. This might negatively influence the virus- receptor binding and thus abrogate the infection (Vincent et al. 2005). As already referred to, a recently published clinical pilot study indicated that hydroxychloroquine treatment was significantly associated with a reduced SARS-CoV-2 viral load (Gautret et al. 2020). These and other small anectodal studies have been challenged and randomized clincal trials have yet to establish the efficacy of hydroxychloroquine for use in Covid-19. Chloroquine/hydroxychloroquine compounds also exert multiple anti-inflammatory effects and have been used for a long time to treat chronic inflammation in lupus and rheumatoid arthritis. The mechanisms for these anti-inflammatory effects are not fully understood, but some have been established and will be outlined here. Chloroquine has been demonstrated to decrease HMGB1 secretion from activated innate immunity cells (Schierbeck et al. 2010). Chloroquine-mediated alkalinization of lysosomes inhibiting HMGB1-caused lysosomal leakage is a plausible mechanism that might be beneficial for COVID-19 treatment to prevent lysosomal leakage (Fig. 2). Intact lysosome function will prevent the activation of multiple proinflammatory cytosolic receptors. Neutrophils exert a central role in the generation of acute lung injury (Rebetz et al. 2018) and acute pancreatitis (Murthy et al. 2019). It is thus relevant to consider that chloroquine has been reported to mediate beneficial treatment effects in a preclinical model of acute pancreatitis by reducing the formation of neutrophil extracellular traps (NETs) (Papayannopoulos 2018). NETs are generated when activated neutrophils release their DNA, histones, HMGB1, and intracellular granule components that together promotes inflammation and tissue damage.

#### Heparin and heparinoid compounds

Heparin is a high affinity HMGB1-binding molecule (Ling et al. 2011; Li et al. 2015; Rouhiainen et al. 2018). The conformation of HMGB1 changes when heparin combines with HMGB1 and this change inhibits the binding of HMGB1 to the surface of activated macrophages (Ling et al. 2011). Heparin-HMGB1 complexes are unable to induce RAGE dimerization that is required for the function of RAGE (Rouhiainen et al. 2018) (Fig. 2). Heparin treatment reduced the lethality in mice exposed to LPS-HMGB1 complexes (Li et al. 2015). Since heparin treatment includes risks of causing potentially dangerous bleeding one might consider to use low-molecular heparin preparations with very low anticoagulant activity but preserved capacity to bind to HMGB1. One such heparinoid compound has been successfully tested clinically in phase I/II studies of *Plasmodium falciparum* malaria disease (Leitgeb et al. 2017). This molecule also prevented neutrophil-induced lung plasma leakage in a murine sepsis model (Rasmuson et al. 2019). As already mentioned, neutrophil-mediated pathology is of central importance in acute lung injury.

#### Thrombomodulin

Thrombomodulin is an endothelial cell thrombin receptor known to convert thrombin into an anticoagulant. Soluble thrombomodulin also binds to HMGB1 and aids the proteolytic cleavage of HMGB1 by thrombin (Abeyama et al. 2005). There is a great number of clinical and preclinical reports of successful thrombomodulin treatment in inflammatory conditions (Ito et al. 2016). Recombinant thrombomodulin is efficaciously used in Japan to treat patients with disseminated intravascular coagulation in sepsis (Yamakawa et al. 2019).

#### Haptoglobin

The major mission of the acute phase protein haptoglobin is to bind and eliminate extracellular hemoglobin, but haptoglobin also captures extracellular HMGB1 (Yang et al. 2016b). Hemorrhage in an inflammatory process will dislocate HMGB1 from haptoglobin and fuel inflammation, since haptoglobin has an exceptionally strong affinity for hemoglobin. Haptoglobin-HMGB1 complexes bind to CD163 on macrophages activating an anti-inflammatory response mediated via production of IL-10 and heme-oxygenase 1 (Yang et al. 2016b). Therapeutic administration of haptoglobin improved septic shock, lung injury, and survival in an experimental pneumonia model (Remy et al. 2018).

Haptoglobin is approved in Japan to treat patients with trauma, burns, and transfusion-related hemolysis.

#### Resveratrol

Resveratrol is a phytoalexin phenol molecule acting as a protective endogenous antibiotic when produced in plants under stress. Resveratrol suppresses TLR4 expression (Yang et al. 2014) and both in vitro and in vivo studies demonstrate that resveratrol activates SIRT1 to reduce HMGB1/TLR4/ MyD88/NF-κB signaling (Le et al. 2019). These results indicate that resveratrol ameliorates inflammation in part via inhibited HMGB1/TLR4-mediated signaling.

#### Statins

Statins are extensively used for treatment of cardiovascular diseases due to their cholesterol-lowering effects, but they also exert beneficial anti-inflammatory functions. These effects are partly brought about by inhibition of both the HMGB1/TLR4- and HMGB1/RAGE-mediated pathways (Liu et al. 2013; Wu et al. 2013; Han et al. 2015; Zhu and Fang 2016; Zhang et al. 2018a) **(**Fig. 2**)**. The expression of HMGB1, RAGE, and TLR4 were all reduced by statin treatment in different vascular inflammatory diseases.

#### Glycyrrhizin

Glycyrrhizin is an active component extracted from licorice plant roots and exerts multiple anti-inflammatory activities including a down-regulation of HMGB1-mediated inflammation. The compound is widely utilized in traditional Chinese medicine to treat inflammatory conditions.

Glycyrrhizin mediates both anti-viral and anti-inflammatory activities (Michaelis et al. 2010; Michaelis et al. 2011; Hoever et al. 2005; Cinatl et al. 2003). Glycyrrhizin has been reported to inhibit in vitro replication of SARS-coronavirus (SARS-CoV), H5N1 influenza A and certain flaviviruses (Michaelis et al. 2010; Cinatl et al. 2003; Crance et al. 2003). Glycyrrhizic derivatives have been developed mediating an increased capacity to inhibit SARS coronavirus replication (Hoever et al. 2005). In addition to the repressed virus replication, glyccyrrhizin inhibited adsorption and penetration of the SARS coronavirus (Cinatl et al. 2003). Glycyrrhizin also attenuated pulmonary inflammation, decreased microvascular permeability and HMGB1 release in an experimental model of LPS-induced acute lung injury (Qu et al. 2019).

#### The cholinergic anti-inflammatory pathway

Acetylcholine mediates potent anti-inflammatory effects by signaling via α7 nicotinic acetylcholine receptors (α7nAChR), a mechanism named “the cholinergic anti-inflammatory pathway” (Andersson and Tracey 2012). These receptors are expressed on alveolar macrophages and lung epithelial cells (Su et al. 2007). Specific α7 nAChR agonist therapy decreased excess lung water, lung vascular permeability, and reduced protein concentration in the bronchoalveolar lavage in a preclinical model of sterile acute injury. Alpha7 nAChR gene-deficient mice expressed a 2-fold increase in excess lung water and lung vascular permeability in the same model (Su et al. 2007). These results indicate that neuronal mechanisms may down-regulate pulmonary inflammation, something that might provide novel therapeutic opportunities. A link between the cholinergic anti-inflammatory system and HMGB1-induced inflammation is that RAGE-mediated endocytosis of HMGB1 complexes with other proinflammatory molecules is restrained by acetylcholine as well as α7nicotinic acetylcholine receptor (α7nAChR)- specific agonists (Yang et al. 2019a) (Fig. 2). An analogous blockade of RAGE/HMGB1-regulated endocytosis has been demonstrated to be mediated by truncated HMGB1 box A protein, an anti-HMGB1 mAb (2G7), and dynamin inhibitors (Yang et al. 2019a).

Acetylcholine signaling via α7nAChR has also been reported to protect against LPS-induced acute lung injury by inhibiting the TLR4/MyD88/NF-κB signaling pathway (Zi et al. 2019).

Acetylcholine-mediated amelioration of inflammation can be accomplished by electrical stimulation of the left vagus nerve (Pavlov et al. 2018). Surgical implantation of vagus nerve pacemakers has demonstrated highly beneficial therapeutical results in rheumatoid arthritis and Crohn’s disease (Koopman et al. 2016; Bonaz et al. 2016). A need for surgery can be circumvented by transauricular vagus nerve stimulation using an external pulse generator, which is an inexpensive device meant for personal use (Hong et al. 2019). Furthermore, transcutaneous vagus nerve stimulation reduced systemic HMGB1 levels and improved survival in an experimental sepsis model (Huston et al. 2007). Another approach to confer cholinergic control over inflammation might be to use the centrally acting acetylcholinesterase inhibitor galantamine (Pavlov et al. 2018). Galantamine is in clinical use for counteracting cognitive impairment in Alzheimer’s disease, but has also been demonstrated to ameliorate inflammation in the metabolic syndrome (Consolim-Colombo et al. 2017).

#### Dexmedetomidine

Dexmedetomidine is a potent α2-adrenergic receptor agonist widely used for sedation in intensive care medicine. The compound also reduces systemic proinflammatory cytokine release through the cholinergic anti-inflammatory pathway via a7nAChR-dependent signaling (Xiang et al. 2014). Adminstration of dexmedetomidine has been demonstrated to increase the discharge frequency of cervical vagus nerves resulting in reduced release of proinflammatory mediators and improved survival in experimental endotoxemia (Xiang et al. 2014). Histological studies of lung sections from LPS-induced lung injury revealed reduced expression of TLR4 and HMGB1 (Meng et al. 2018).

Furthermore, combined dexmedetomidine-ketamine treatment in endotoxemic rats mitigated pulmonary inflammatory response induced by ventilator-induced lung injury (Yang et al. 2011).

#### Ketamine

Ketamine is another extensively used pharmacological substance in anesthesia and is judged as safe and to facilitate hemodynamically stable anesthesia or sedation. It also mediates anti-inflammatory functions including inhibition of HMGB1 secretion from activated macrophages (Zhang et al. 2014). Furthermore, in preclinical studies ketamine has been shown to attenuate sepsis-induced acute lung injury via a functional down-regulation of the HMGB1-RAGE pathway (Zhang et al. 2018b) (Fig. 2). Ketamine reduced the recruitment of neutrophils and monocytes into the inflamed lungs, and diminished myeloperoxidase activity and the expression of HMGB1 and TLR4 (Li et al. 2016; Qin et al. 2015). Since COVID-19 patients with severe ARDS may need a long period with ventilator support, ketamine could be considered to be incorporated into the sedation protocol for these patients.

## Conclusions

The highlights of the review are the following:

- The function of HMGB1 for innate immunity is as important as MHC molecules for adaptive immunity. Each carries and presents antigens, although the systems operate in opposite directions. An MHC antigen binds its peptide intracellularly before it expresses the complex on the cell surface to be recognized by a T cell receptor. HMGB1 catches danger molecules extracellularly and brings the complexes intracellularly for destruction. However, a high intralysosomal HMGB1 concentration disrupts the lysosomal membrane allowing danger molecule access to cytosolic receptors mediating inflammation.
- Exaggerated host inflammatory response is a major cause of lung injury and subsequent mortality in many severe pulmonary inflammatory conditions including COVID-19. A hyperexcited HMGB1-RAGE axis can be expected in COVID-19 since necrotic respiratory epithelial cells contribute with large amounts of extracellular HMGB1, and its cognate receptor RAGE is constitutively abundantly expressed in the lungs. Once initiated, inflammation will be intensified via HMGB1 secretion from innate immunity cells causing further RAGE and TLR4 upregulation.
- Arterial HMGB1 levels are considerably lower than those in venous blood and we propose that this connects systemic HMGB1 biology to lung physiology. HMGB1-danger molecule complexes are transported via RAGE to the endolysosomal compartment. Proper lysosomal degradation of these complexes is mandatory for pulmonary function. Increased intralysosomal HMGB1 concentrations cause lysosomal destabilization allowing danger molecules access to potent cytoplasmic receptors causing inflammation that can be lethal.
- HMGB1 acts upstream of the proinflammatory cytokine cascade (Andersson et al. 2000).
- HMGB1 is a DNA- and RNA-binding molecule and HMGB1 has been demonstrated to function as a gene delivery agent. There is a risk that HMGB1 might attach to viral RNA and bring it to the cytosol via the RAGE-lysosomal pathway. This would implicate that there could be an additional pathway apart from the angiotensin-converting enzyme 2 receptors enabling intracellular virus replication.
- A substantial number of preclinical studies demonstrates that HMGB1 antagonists ameliorate severe pulmonary inflammation regardless of infectious or sterile origin. A further study of a therapeutic use of HMGB1-specific antagonists is warranted. Means to prevent HMGB1-danger molecule complexes from binding to RAGE should be sought for.

## Data Availability

Not applicable.

## Abbreviations

HMGB1: High mobility group box 1 protein
COVID-19: Coronavirus disease 2019
SARS-CoV-2: Severe acute respiratory syndrome coronavirus 2
DAMP: Damage-associated molecular pattern
PAMP: Pathogen-associated-molecular-pattern
ARDS: Acute respiratory distress syndrome
RAGE: Receptor for advanced glycation end products
TLR4: Toll-like receptor 4
MD-2: Myeloid differentiation factor 2
LPS: Lipopolysaccharide
HRSV: Human respiratory syncytial virus
ICU: Intensive care unit
AIM2: Absent in melanoma 2
cGAS: Cyclic GMP-AMP synthase
RIG-I: Retinoic acid-inducible gene I
SIRT1: Sirtuin 1
